# Glucose concentration in capillary blood of dairy cows obtained by a minimally invasive lancet technique and determined with three different hand-held devices

**DOI:** 10.1186/s12917-016-0662-3

**Published:** 2016-02-24

**Authors:** B. Mair, M. Drillich, D. Klein-Jöbstl, P. Kanz, S. Borchardt, L. Meyer, I. Schwendenwein, M. Iwersen

**Affiliations:** Department for Farm Animals and Veterinary Public Health, Clinical Unit for Herd Health Management in Ruminants, University Clinic for Ruminants, University of Veterinary Medicine Vienna, 1210 Vienna, Austria; FirstFarms Slovakia, 900 68 Plavecký Štvrtok, Slovakia; Department for Pathobiology, Central Clinical Pathology Unit, University of Veterinary Medicine Vienna, 1210 Vienna, Austria; Current address: Clinic for Animal Reproduction, Faculty of Veterinary Medicine, Freie Universität Berlin, Berlin, Germany

**Keywords:** Dairy cow, Glucose, Capillary blood, Hand-held meter

## Abstract

**Background:**

Dairy cows have a massive demand for glucose at the onset of lactation. A poor adaption to this period leads to an excessive negative energy balance with an increased risk for ketosis and impaired animal health and production.

Besides the measurement of ketones, analysing the glucose concentration in blood is reported as helpful instrument for diagnosis and differentiation of ketosis. Monitoring metabolic parameters requires multiple blood sampling. In other species, new blood sampling techniques have been introduced in which small amounts of blood are rapidly analysed using electronic hand-held devices.

The objective of this study was to evaluate the suitability of capillary blood for blood glucose measurement in dairy cows using the hand-held devices FreeStyle Precision (FSP, Abbott), GlucoMen LX Plus (GLX, A. Menarini) and the WellionVet GLUCO CALEA, (WGC, MED TRUST). In total, 240 capillary blood samples were obtained from dry and fresh lactating Holstein-Friesian cows. Blood was collected from the skin of the exterior vulva by using a lancet. For method comparison, additional blood samples were taken from a coccygeal vessel and analyzed in a laboratory. Glucose concentrations measured by a standard laboratory method were defined as the criterion standard.

**Results:**

The Pearson correlation coefficients between the glucose concentrations analyzed in capillary blood with the devices and the reference were 73 % for the FSP, 81 % for the GLX and 41 % for the WGC. Bland-Altman plots showed biases of −18.8 mg/dL for the FSP, -11.2 mg/dL for the GLX and +20.82 mg/dL for the WGC. The optimized threshold determined by a Receiver Operating Characteristics analysis to detect hyperglycemia using the FSP was 43 mg/dL with a sensitivity (Se) and specificity (Sp) of 76 and 80 %. Using the GLX and WGC optimized thresholds were 49 mg/dL (Se = 92 %, Sp = 85 %) and 95 mg/dL (Se = 39 %, Sp = 92 %).

**Conclusions:**

The results of this study demonstrate good performance characteristics for the GLX and moderate for the FSP to detect hyperglycemia in dairy cows using capillary blood. With the study settings, the WGC was not suitable for determination of glucose concentrations.

## Background

Glucose is an essential nutrient for all higher organisms. Certain vitally important cells and tissues like the brain, erythrocytes and the mammary gland are dependent on the energy provided by glucose. Hence, it is important that the concentration of glucose in the blood is kept at a physiological level [1]. Ruminants and particularly dairy cows have a special position with respect to the glucose metabolism, because of the massive demand of their mammary gland for glucose at the onset of lactation and the permanent alteration between pregnancy and lactation. Furthermore, most of the glucose is generated by gluconeogenesis using volatile glucogenic precursors (e.g. propionate, lactate, alanine, valerate and isobutyrate) [2].

Oikonomou et al. [3] reported a significant influence of the glucose concentration on reproductive performance, as glucose is essential as major energy source for ovarian cyclicity [4]. Additionally, a study by Galvão et al. [5] has shown that cows suffering from uterine infections within the first 3 weeks after parturition had a decreased polymorphonuclear neutrophils (PMN) glycogen concentration and hence, a decrease in PMN functions. It was reported that the PMN glycogen concentration is positively associated with the blood glucose concentration. Furthermore, Garverick et al. [6] found greater blood glucose concentration in cows that conceived at first insemination in comparison with unsuccessfully bred cows, and hypothesized that blood glucose concentration within the first 3 weeks after parturition can be used as predictor for the likelihood of pregnancy after first insemination.

For most tissues, the supply and utilization of nutrients is regulated by hormones. Also in Ruminants insulin is a key player in the energy metabolism, influencing various metabolic processes in muscles, adipose tissue, liver and the mammary gland [7]. Ruminants are reported to suffer from ‘insulin resistance’ (IR) at the end of gestation and during early lactation [8–10]. The phenomenon of IR is characterized by a less than normal biological response to a physiological concentration of insulin, as a result of decreased sensitivity of the tissues, a decreased responsiveness to insulin or a combination of both [11].

Insulin resistance at the end of gestation and at early lactation are considered as physiologic mechanism to ensure the glucose and hence energy supply for the gravid uterus and lactating mammary gland. However, IR can transgress into a pathologic state, leading to ketosis for instance with chronically increased concentrations of ß-hydroxybutyric acid (BHBA) in cows [2]. From an aetiological point of view two different types of ketosis exist: First the hypoglycaemic-hypoinsulinaemic form, ‘Type I’-ketosis, which occurs three to 6 weeks after calving in high-yielding cows where the energy demand in the udder exceeds the capacity of gluconeogenesis. Second, the hyperglycaemic-hyperinsulinaemic form, ‘Type II’-ketosis exists, which occurs earlier in lactation as a consequence of overfeeding the animals in the dry period [12]. Besides the measurement of the BHBA concentration, analysing the glucose concentration in blood is reported as helpful instrument for diagnosis and especially differentiation between these two types of ketosis [13, 14], although Herdt [15] described the glucose concentration as an insensitive measure of the energy status of an animal.

Monitoring metabolic parameters requires multiple blood sampling, which is usually done by repeated venepuncture in cattle. This procedure requires handling or restraining of the animal, which may result in stress-induced hyperglycemia [16]. In other species, new techniques have been introduced in which small amounts of blood are rapidly analysed for glucose concentrations using electronic hand-held devices [17–19]. Applying these minimally invasive techniques to obtain capillary blood in dairy cattle might be beneficial in generating reliable and valid glucose concentrations.

The primary objective of this study was to test whether capillary blood can be used to determine the glucose concentrations in dairy cows using minimally invasive techniques. A second objective was to evaluate test characteristics of three commercially available hand-held devices for determination of blood glucose concentrations. Therefore a capillary blood drop, obtained from the skin of the exterior vulva, was tested with three different hand-held devices. Additionally, glucose concentrations in coccygeal blood were analysed in a laboratory to be used as ‘criterion standard’ [20] and analysed with the hand-held devices, too to distinguish between the effect of the device and the type of blood sample used for the on-site test.

## Methods

The study was approved by the institutional ethics committee of the University of Veterinary Medicine, Vienna (04/12/97/2013; date of approval 17 December 2013) according to the Good Scientific Practice guidelines and the national authority according to § 26 of Law for Animal Experiments [Tierversuchsgesetz 2012 – (TVG 2012); BMWF GZ 68.205/0007-II/3b/2014] as well as by the Slovakian Regional Veterinary Food Administration (428/2014) and was conducted between March and April 2014. A written informed consent was obtained from the farm owner to collect samples and to publish the study results. The farm involved in the study adheres to a high standard of veterinary care based on standard operating procedures.

### Animals

A commercial dairy farm in Slovakia with approximately 2700 Holstein-Friesian cows and additional youngstock was chosen as study site. The dairy herd was kept in free stall barns with rubber mats on concrete floors. Before calving the cows were moved in a free stall barn with straw beddings. A total mixed ration was fed twice per day and pushed up frequently. The average energy corrected milk yield (based on 4.0 % butterfat and 3.4 % protein) was 9165 kg in 2013.

A sample size calculation (type I error α = 0.05, type II error β = 0.2) was performed to detect a maximum irrelevant difference in the glucose concentration of 3 mg/dL between the methods evaluated in the study, resulting in 182 animals needed. To compensate for potential data losses due to necessary exclusions because of pre-analytical or analytical problems 240 animals were enrolled in the study. Animals of all lactations within 2 weeks ante-partum up to 4 weeks post-partum were eligible for enrollment.

### Sampling procedures

**Fig. 1:**
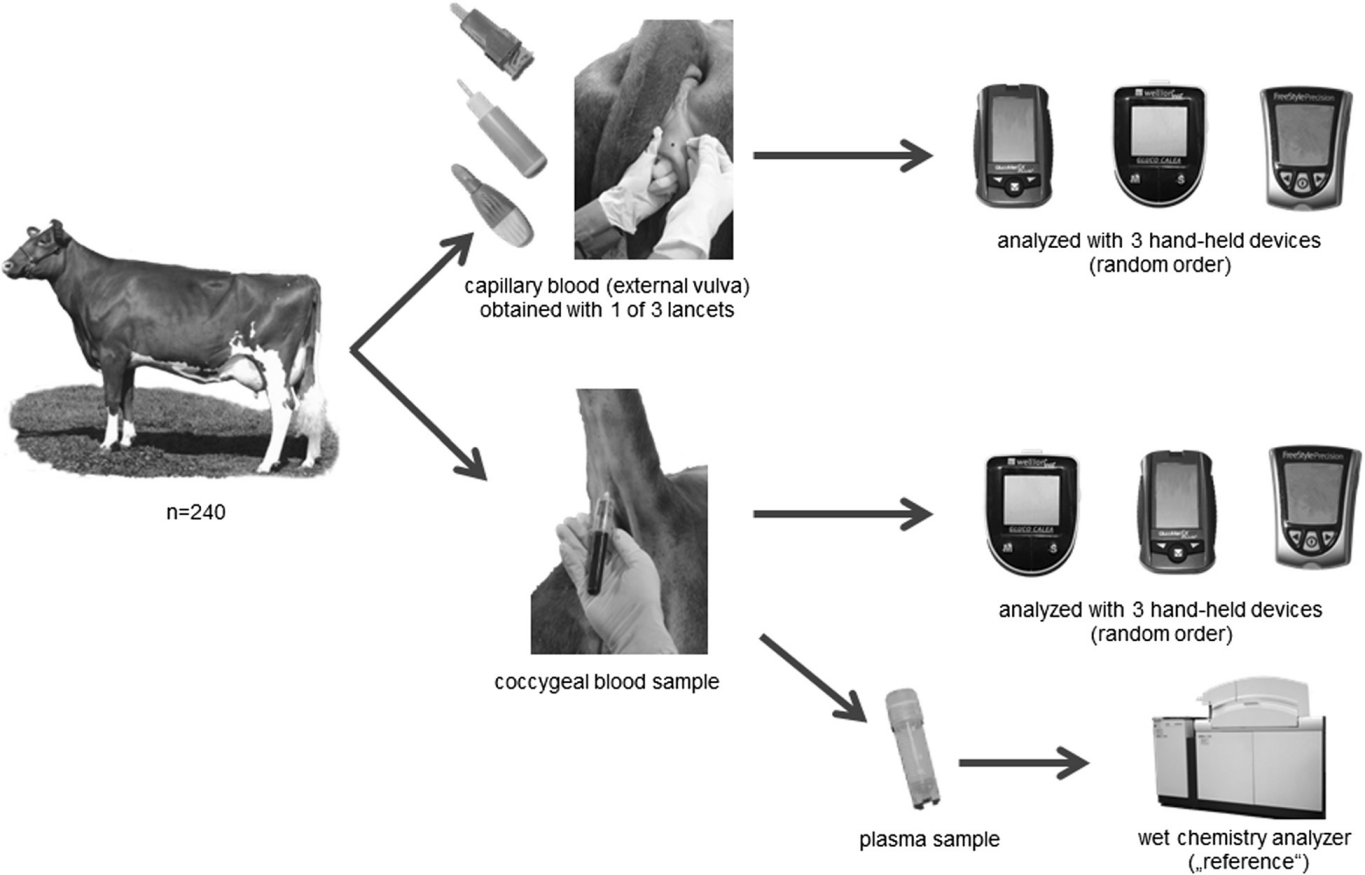
Sampling procedures. In total, 240 dairy cows were enrolled. Capillary blood was obtained from the external vulva by using 1of 3 lancet types. The glucose concentrations in the capillary blood drop as well as in a coccygeal blood sample were analyzed each, by using 3 different hand-held devices. Plasma obtained from the coccygeal blood sample was analyzed for glucose in the laboratory of the University using a wet chemistry analyzer and was regarded as “reference” in the study

The sampling procedures are illustrated in Fig. 1. To obtain capillary blood, the skin of the exterior vulva was dry-cleaned and punctured with one of three different available lancets, in random order. The lancets used in the study [Microtainer Contact-Activated Lancet (MT, Becton-Dickinson), SafetyLancets special (SL, MED TRUST Handelsges.m.b.H.), MiniCollect Safety Lancets (MC, Greiner Bio-One International AG)] have a penetration depth of 2 mm with differing blade widths between 0.8 (SL) to 1.5 mm (MT and MC). If the volume of the obtained blood drop was insufficient for determination of the glucose concentration, another puncture was made. The quantity of punctures was recorded on a pre-assembled data capture form. Three different electronic hand-held meters [FreeStyle Precision, (FSP, Abbott GmbH & Co. KG), GlucoMen LX Plus, (GLX, A. Menarini GmbH), WellionVet GLUCO CALEA, (WGC, MED TRUST Handelsges.m.b.H.)] and the associated electrochemical test strips [FreeStyle Precision blood glucose, (Abbott Diabetes Care), GlucoMen LX Sensor, (A. Menarini GmbH), WellionVet GLUCO CALEA test strips, (MED TRUST Handelsges.m.b.H.)] were used in the study to determine the blood glucose concentration. The WGC was already validated for use in dogs, cats and horses by using preprogrammed species-specific chips, offered with each batch of test strips. Because of best possible match in terms of red blood cell parameters, the chip initially designed for use in cats was chosen for measurements in this study.

### Operating principle of the hand-held devices and laboratory analyses

The test principle of all 3 hand held devices is based on an amperometric biosensor technology. The meter systems consist of two components: The meter itself processes the data by applying integrated algorithms and presents the results on an integrated display. The associated biosensor test strips are designated to analyze a small amount of a blood sample (i.e. for the GLX: 0.3 μL; WGC: 0.5 μL; FSP: 0.6 μL) by initiating further enzymatic reactions.

After the application of the blood on the sensor, an electrochemical reaction starts using either glucose dehydrogenase (FSP) or glucose oxidase (GLX and WGC) as catalysator. The enzymes are oxidoreductases and oxidize glucose to gluconolactone. By this, electrons from the glucose are transferred to the oxidized form of a mediator, thereby converting it to the reduced form. By re-oxidation of the mediator electrons are transferred to the electrode surface. The resulting current is monitored by the meter and directly proportional to the glucose concentration in the blood sample. Using meter specific software algorithms, the current is converted into a measure of the glucose concentration which is displayed as digital value.

The sensors of the 3 hand-held devices were directly dipped onto the surface of the capillary blood drop. After approximately 5 s each, the blood glucose concentrations were presented on the display of the devices.

Besides the manufacturer’s intended and certified use of testing capillary blood, the devices were used “off-label” for measuring the glucose concentration in venous and/or arterial blood. For this, an additional blood sample was drawn from a coccygeal vessel using a blood-collection tube system (Vacuette, Greiner Bio-One GmbH) consisting of a Sodium Fluorid vacuum tube (Vacuette, FX Sodium Fluoride, Greiner Bio-One GmbH) and a 20-gauge needle (Vacuette 0.9 × 38 mm, Greiner Bio-One GmbH). Blood obtained from the coccygeal vessel was tested as well with all three devices as previously described. Approximately 2 h after collection, the coccygeal blood samples were centrifuged at 2200 × *g*, at a temperature of 18 °C for 5 min. (Eppendorf Centrifuge 5804, Eppendorf AG). Supernatant plasma was split into two aliquots in microtubes of 2 ml each (Microtube, Sarstedt), as reference and back-up sample and were stored at −18 °C until further analyses. The glucose concentration of this plasma sample was analyzed at the Central Clinical Pathology Unit (CCPU) of the University of Veterinary Medicine, Vienna and was considered as the reference value (i.e. the criterion standard) in the present study. Plasma was analyzed with an automated wet chemistry analyzer Cobas 6000/c501 (Roche Diagnostics GmbH, Vienna, Austria) using a colorimetric hexokinase method. Hexokinase catalyzes the phosphorylation of glucose to glucose-6-phosphate and adenosine-diphosphate by ATP. This reaction is coupled with an NADP colorimetric indicator system.

To evaluate the intra-assay variability of the analyses performed at the CCPU, a subset of 20 aliquots of plasma, obtained from a blood sample of one cow, were randomly placed among the samples obtained from the study animals. Furthermore, intra- and inter-assay coefficients of variations (CV) were calculated for each specific hand-held device. For this, three blood samples with different glucose concentrations based on FSP measurements with low (30 mg/dL), medium (56 mg/dL) and high (70 mgl/dL) glucose concentrations were tested ten times with one device (intra-assay) and additionally with ten different devices of the same type (inter-assay).

### Statistical analyses

The data were analyzed using SPSS statistics for Windows (Version 20.0; IBM Deutschland GmbH) and BiAS for Windows (Version 10.06; Epsilon-Verlag). Data were tested for normal distribution using the Kolmogorov-Smirnov-Test. For each tested hand-held device, descriptive statistics were estimated for the glucose concentrations analyzed in capillary and coccygeal blood. Additionally, the Pearson correlation coefficients were calculated between the reference and the glucose concentrations measured with each specific device in capillary and coccygeal blood. The level of significance for all statistical tests was set at *α* = 0.05.

A Passing-Bablok regression [21] was performed to compare the glucose concentration in the reference sample with the concentrations measured with the three hand-held devices in capillary and coccygeal blood. For this, the slopes and the intercepts of the regression lines and their 95 % confidence intervals were determined. The intercept (*a*) reflects the constant differences and the slope (*b*) the proportional differences between the two methods. If the confidence interval for the intercept includes the value 0, no constant difference between the two methods exists. If the interval for the slope includes the value 1, no proportional difference occurs. If neither a constant nor a proportional difference could be observed, both methods can be used interchangeably [22]. In addition, the agreement between the reference and the hand-held meters were graphically depicted using the method as reported by Bland and Altman [23].

Based on the glucose concentrations analyzed at the CCPU, samples were classified as hypo- (<40 mg/dL), normo- (40–60 mg/dL) or hyperglycemic (>60 mg/dL). According to these classifications sensitivities (Se) and specificities (Sp) for each hand-held device to detect hypo- and hyperglycemia were calculated. To determine optimized thresholds to identify hypo- and hyperglycemia using the hand-held meters, Receiver Operating Characteristics (ROC) analyses were performed. The closer the resulting graph of the ROC analysis is to the left upper angle of the coordinate system, the greater is the accuracy of the test [24]. The resulting area under the ROC curve (AUC) is a measure of the discriminatory power of a test to identify animals as normoglycemic and hypo- or hyperglycemic, respectively. An AUC of 1 represents a perfect test; an AUC of 0.5 and below represents a worthless test. ROC analyses were based on maximizing the Youden-Index (*J*), calculated as (Se + Sp - 1). The *J* reflects the number of all correctly identified outcomes and can range from 0 to 1. A value close to1 indicates a valuable diagnostic test, whereas a value close to 0 implies a worthless test [25].

## Results

The mean glucose concentration of the 20 samples taken from one cow to evaluate the intra-assay variability of measurements at the CCPU was 44.45 ± 0.83 mmol/L, resulting in a CV of 1.86 %.

In total, 240 Holstein-Friesian cows were tested. Thirty four (14.2 %) of the animals were in first lactation, 97 (40.4 %) in second lactation, and 109 (45.4 %) in third or greater lactation. All animals were sampled between 21 days ante-partum and 29 days post-partum (median 7 days in milk; 25 % Percentile 3 and 75 % Percentile 13), resulting in 199 (83 %) samples from lactating cows and 41 (17 %) samples from dry cows.

The reference samples comprised two samples (0.8 %) classified as hypoglycemic (glucose < 40 mg/dL), 149 samples (62.2 %) as normoglycemic (glucose between 40 to 60 mg/dL) and 89 samples (37.1 %) as hyperglycemic (glucose > 60 mg/dL). All lancets used in the study were eligible for capillary blood specimen collection, and only small numerical differences could be observed in the total number of incisions needed. Capillary blood could be obtained with first incision in 94 % (*n* = 75) using the SL, 96 % (*n* = 77) using the MT and in 96 % (*n* = 77) using the MC lancet. An additional second (and third) incision was necessary for the MT in three (two) cases, using the SL in two (zero) and the MC in three (zero) cases. Measurements of four (1.7 %) capillary blood samples using the GLX device resulted in an “E4”-failure. According to the manufacturer’s instructions this error message indicates a “damaged sensor, incorrect application or quantity of the blood sample”. Additionally, an “E3”-failure was shown by the FSP in two (0.8 %) measurements of capillary blood, indicating “a test error or glucose concentrations below the detection limit”. These six measurements were repeated.

Descriptive parameters for the analyzed glucose concentrations are presented in Table 1. The Pearson correlation coefficients between the glucose concentrations analyzed in capillary blood using the hand-held meters and the reference were 73.3 % for the FSP, 80.5 % for the GLX and 41.2 % for the WGC (*P* < 0.01, for all three devices). Using coccygeal blood, the corresponding correlation coefficients were 86.6 % for the FSP, 78.8 % for the GLX and 50.5 % for the WGC (*P* < 0.01, for all three devices).

**Table 1:** Descriptive statistics of the glucose concentrations analyzed in coccygeal and capillary blood using three different hand-held devices as well as in plasma analyzed at the laboratory (reference)

Parameter	Plasma^a^	Capillary blood			Coccygeal blood		
	Laboratory	FSP^b^	GLX^c^	WGC^d^	FSP	GLX	WGC
Number of samples (n)	240	240	240	240	240	240	240
Mean (mg/dL)	58.17	39.56	46.96	78.99	53.28	48.18	82.86
SD (mg/dL)	10.48	11.65	10.75	16.69	12.33	10.48	17.59
Median (mg/dL)	58.00	38.00	46.00	79.00	53.00	49.00	83.00
Interquartile range (mg/dL)	12.50	18.00	16.00	26.00	16.00	13.00	26.00

^a^obtained from coccygeal blood (reference)

^b^FSP: FreeStyle Precision, (Abbott GmbH & Co. KG, Wiesbaden, Germany)

^c^GLX: GlucoMen LX Plus (A. Menarini GmbH, Vienna Austria)

^d^WGC: WellionVet GLUCO CALEA (MED TRUST Handelsges.m.b.H., Marz, Austria)

**Fig. 2:**
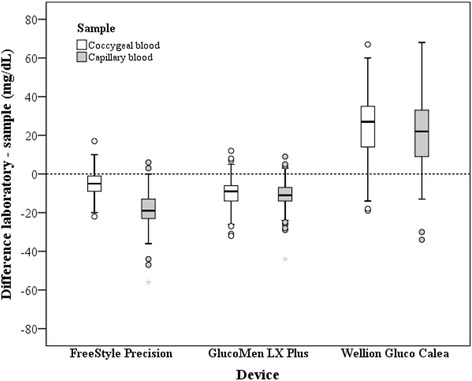
Parallel boxplots showing the differences between the glucose concentrations in plasma analyzed at the laboratory (reference) and the concentrations measured with 3 different hand-held devices in either whole blood obtained from a tail vessel or in capillary blood. The heavy black line inside each box marks the median (50th percentile); lower and upper hinges mark the 25th and 75th percentiles. Whiskers end at the smallest and largest statistical values that are not outliers; outliers and extreme values are designated by o and *

The differences between the glucose concentrations analyzed in plasma at the laboratory and the concentrations measured with the hand-held devices either in whole blood obtained from a coccygeal vessel or in capillary blood are presented in Fig. 2. The glucose concentrations analyzed with the FSP and the GLX in the capillary blood as well as in the coccygeal blood resulted in smaller glucose concentrations compared with the laboratory results in plasma. Measuring the glucose concentrations using the WGC overestimated the laboratory results.

**Fig. 3:**
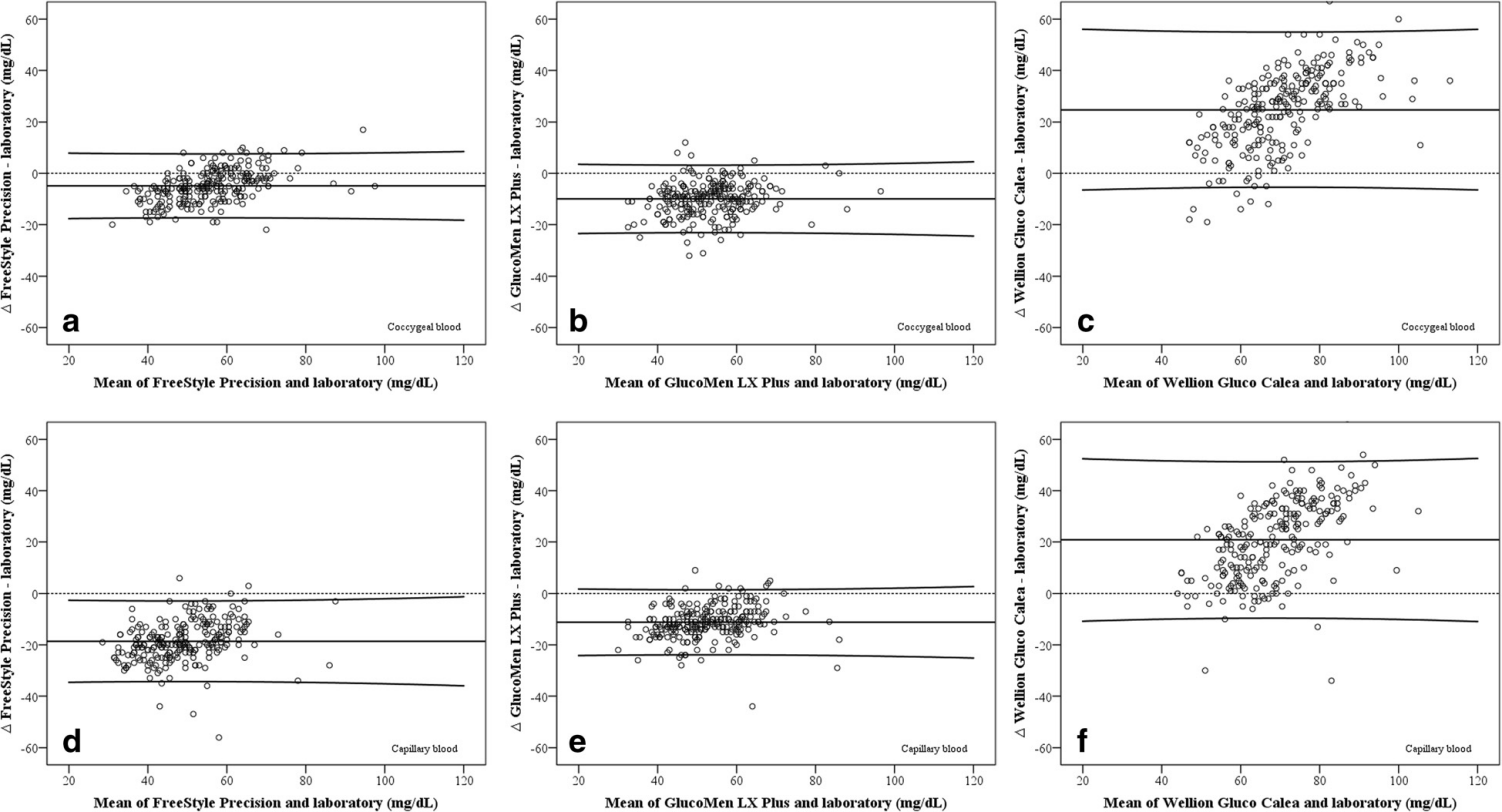
Bland-Altman plots of differences in glucose concentrations measured with the FreeStyle Precision (panels **a** and **d**), GlucoMen LX Plus (panels **b** and **e**) and Wellion GLUCO CALEA (panels **c** and **f**) hand-held devices in coccygeal (*top*) or capillary (*bottom*) blood and the reference test against their mean. The solid line in the middle represents the mean; the solid upper and lower lines represent the mean ± 2 SD

Bland-Altman plots (Fig. 3) between the reference test and coccygeal blood demonstrated a negative bias of −4.89 ± 6.31 mg/dL for the FSP and −9.98 ± 6.67 mg/dL for the GLX, whereas a positive bias for the WGC of +24.64 ± 15.19 mg/dL was determined. Using capillary blood for testing, biases of −18.80 ± 7.96 mg/dL for the FSP, −11.20 ± 6.42 mg/dL for the GLX and +20.82 ± 15.41 mg/dL for the WGC were detected (Table 2 and Fig. 3).

**Table 2:** Differences between the glucose concentrations analyzed by three different hand-held devices and the laboratory results using the Bland-Altman analysis method and Passing-Bablok regression analysis

Device^a^	Capillary blood						Coccygeal blood					
	Passing-Bablok				Bland-Altman		Passing-Bablok				Bland-Altman	
	Slope (*b*)	CI_95_^b^ for *b*	Intercept (*a*)	CI_95_ for *a*	Bias (*d*)	SD of *d*	Slope (*b*)	CI_95_ for *b*	Intercept (*a*)	CI_95_ for *a*	Bias (*d*)	SD of *d*
FSP	0.73	0.67–0.80	29.30	26.70–31.67	−18.80	7.96	0.72	0.66–0.78	19.43	16.28–22.33	−4.89	6.31
GLX	0.82	0.75–0.88	19.57	16.67–23.00	−11.20	6.42	0.88	0.80–1.00	14.93	9.00–19.00	−9.98	6.67
WGC	0.41	0.34–0.50	24.53	17.50–30.64	20.82	15.41	0.40	0.34–0.47	23.00	17.16–28.09	24.64	15.19

^a^FSP: FreeStyle Precision, (Abbott GmbH & Co. KG, Wiesbaden, Germany), GLX: GlucoMen LX Plus (A. Menarini GmbH, Vienna, Austria), WGC: WellionVet GLUCO CALEA (MED TRUST Handelsges.m.b.H, Marz, Austria)

^b^CI_95_: 95 % confidence interval

To compare the specific results of the hand-held devices with the reference, a Passing-Bablok regression was performed (Table 2). Using the GLX for measurements in coccygeal blood, the confidence interval for the slope includes the value 1, but the confidence interval for the intercept did not include 0. Hence, no proportional but a constant difference was detected. Using the FSP and the WGC in coccygeal blood, a proportional and a constant difference exist. Similar findings for measurements using the three hand-held devices in capillary blood were detected.

**Fig. 4:**
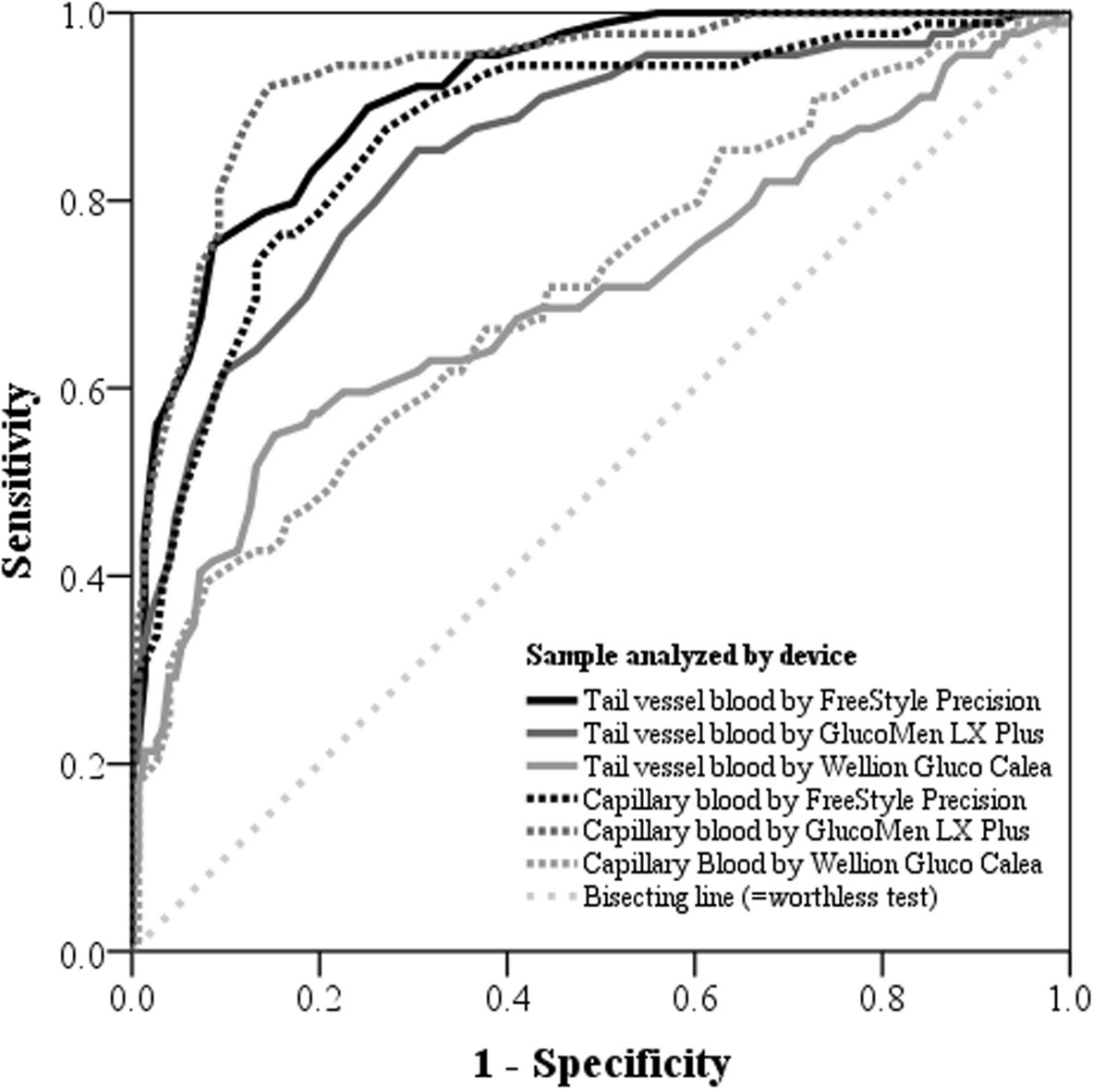
Receiver operating characteristics curves for three different hand-held devices for diagnosis of hyperketonemia either in tail vessel or capillary blood, based on laboratory glucose concentration in plasma > 60 mg/dL as threshold for hyperketonemia

Receiver Operating Characteristics analyses were intended to calculate the optimal thresholds to differentiate between normo- and hyperglycemia using the electronic hand-held devices. Because only two reference samples were classified as hypoglycemic, no reliable ROC analyses for those samples were possible. Hence, the results are not presented. Detailed results of the ROC analyses for the three different hand-held devices to detect hyperglycemia as well as further test characteristics are presented in Table 3 and Fig. 4.

**Table 3:** Thresholds and corresponding test characteristics of 3 different hand-held meters to detect hyperglycemia in capillary and coccygeal blood based on Receiver Operating characteristics (ROC) analysis using a laboratory plasma glucose concentration of ≥ 60 mg/dL as reference

Device^a^	Capillary blood					Coccygeal blood				
	Optimized device threshold^b^ [mg/dL]	AUC^c^ [%(CI_95_^d^)]	Se^e^ [%(CI_95_)]	Sp^f^ [%(CI_95_)]	Youden-Index^g^	Optimized device threshold^c^ [mg/dL]	AUC [%(CI_95_)]	Se [%(CI_95_)]	Sp [%(CI_95_)]	Youden-Index
FSP	43	87.4 (82.5–91.3)	76.4 (66.2–84.8)	84.1 (77.3–89.5)	0.61	59	91.7 (87.5–94.9)	75.3 (65–83.8)	91.4 (85.7–95.3)	0.67
GLX	49	93.4 (89.5–96.2)	92.1 (84.5–96.8)	85.4 (78.8–90.6)	0.78	49	85.2 (80.1–89.5)	85.4 (76.3–92)	69.5 (61.5–76.8)	0.55
WGC	95	70.5 (64.3–76.3)	39.3 (29.1–50.3)	92.1 (86.5–95.8)	0.31	94	70.0 (63.8–75.7)	55.1 (44.1–65.6)	84.8 (78–90.1)	0.40

^a^FSP: FreeStyle Precision, (Abbott GmbH & Co. KG, Wiesbaden, Germany), GLX: GlucoMen LX Plus (A. Menarini GmbH, Vienna, Austria), WGC: WellionVet GLUCO CALEA (MED TRUST Handelsges.m.b.H, Marz, Austria)

^b^based on Receiver Operating Characteristics (ROC) analyses

^c^AUC: area under the receiver operating characteristics (ROC) curve

^d^CI_95_: 95 % confidence interval

^e^Se: sensitivity

^f^Sp; specificity

^g^Youden-Index = (Se + Sp – 1)

The calculated AUCs for measurements in capillary and coccygeal blood indicate that the GLX (93 and 85 %) and the FSP (87 and 92 %) were more eligible in determining hyperglycemia than the WGC (71 and 70 %). With a calculated AUC of 93 % and a Youden-Index of 78 % the GLX was the most capable device to detect hyperglycemia in capillary blood. Sensitivities and specificities using the optimized thresholds for all three devices are shown in Table 3. Identifying hyperglycemia in capillary blood, Se was greatest for the GLX (93 %), however Sp was greatest for the WGC (92 %).

The calculated inter- and intra-assay CV for analyzing the glucose concentration using the different devices are presented in Table 4. The average inter- and intra-assay CVs were acceptable for the FSP with 4.2 and 5.2 %, but less satisfying for the GLX with 19.9 and 14.4 %, and for the WGC with 12.6 and 15.7 %.

**Table 4:** Intra-assay and inter-assay coefficients of variation for hand-held meters FreeStyle Precision (FSP), GlucoMen LX Plus (GLX) and WellionVet GLUCO CALEA (WGC) within low, medium and high glucose concentrations

Parameter	Glucose concentration^a^											
	FSP^c^				GLX^d^				WGC^e^			
	Low	Medium	High	Average	Low	Medium	High	Average	Low	Medium	High	Average
Intra-assay												
Mean [mg/dL]	26.80	46.60	63.50	45.64	28.90	57.40	73.30	53.20	109.50	122.20	119.60	117.10
SD [mg/dL]	1.99	2.07	2.27	2.11	2.64	11.30	10.58	8.17	19.70	20.20	15.20	18.37
CV^b^ [%]	7.43	4.44	3.57	5.15	9.13	19.69	14.43	14.42	17.99	16.53	12.71	15.74
Inter-assay												
Mean [mg/dL]	26.40	48.40	66.10	46.70	40.00	58.70	74.50	57.73	93.40	113.30	114.30	107.00
SD [mg/dL]	1.58	1.65	2.18	1.80	8.94	12.96	11.35	11.08	14.39	16.53	8.94	13.29
CV^b^ [%]	5.98	3.41	3.30	4.23	22.35	22.08	15.23	19.89	15.41	14.59	7.82	12.61

^a^Based on measurements using FreeStyle Precision (low = 30 mg/dL; medium = 56 mg/dL; high = 70 mg/dL)

^b^ Coefficient of variation = SD × 100/mean

^c^FSP = FreeStyle Precision (Abbott GmbH & Co. KG, Wiesbaden, Germany)

^d^ GLX = GlucoMen LX Plus (A. Menarini GmbH, Vienna, Austria)

^e^ WGC = WellionVet GLUCO CALEA (MED TRUST Handelsges.m.b.H, Marz, Austria)

## Discussion

To our knowledge, this is the first study evaluating the suitability of the three hand-held meters to determine the glucose concentration in capillary blood in dairy cows. Using a minimally invasive technique to obtain capillary blood, as already used in small animal medicine [17–19], is considered as useful tool for monitoring the energy metabolism in dairy cows [26]. Additionally, measurements of the glucose concentrations in cows already suffering from hyperketonemia is beneficial to gain further insights in the type of existing ketosis [13] in order to optimize the animals’ treatment. For these purposes, a reliable and accurate test system is essential.

Based on laboratory thresholds, the number of plasma samples (*n* = 2, 0.8 %) classified as hypoglycaemic was low. This was not expected because several cows were sampled in the early lactation where a negative energy balance is expected. It can be speculated if these results support the hypothesis of a physiological insulin-resistance in dairy cows [12]. Hence, further studies are planned to investigate the usefulness of population based clinical decision limits to detect a hypoglycaemic condition in cows during the transition period.

Whereas the FSP and the GLX were already validated for BHBA measurements to diagnose subclinical ketosis in dairy cows [27, 28], the WGC has never been used for any studies in bovines. It should be mentioned, that the chip used in this study was intended for use in cats and not certified by the manufacturer for use in bovines. The suitability of different hand-held devices to analyze glucose concentrations in blood obtained from the jugular vein or a coccygeal vessel in dairy cows were already evaluated by others [13, 29, 30]. For the FreeStyle Precision Xtra device (Abbott Diabetes Care Inc.), a previous version of the FSP, Wittrock et al. [30] determined a Pearson correlation coefficient between coccygeal whole blood samples and the corresponding plasma glucose concentration analysed at a laboratory of 95 % with a mean difference of -0.03 ± 1.96 mmol/L (equivalent to −0.54 ± 35 mg/dL). Voyvoda and Erdogan [13] determined a correlation coefficient between the glucose concentration in jugular whole blood analyzed with the Optium Xceed device (Abbott Diabetes Care) and its corresponding laboratory plasma concentration of 63 % with a mean difference of 0.27 mmol/L (4.86 mg/dL). Evaluating the One Touch II (Lifescan Inc) Roeder et al. [29] reported a correlation coefficient of 94 % with a mean difference of 12.95 mg/dL for samples obtained either from the jugular vein in calves, or by coccygeal venepuncture in cows and their plasma glucose concentrations, analysed at a laboratory. The results of our study, using the FSP and the GLX devices for analysing the glucose concentration in coccygeal blood showed similar deviations from the laboratory results as reported by Roeder et al. [29] and Voyvoda and Erdogan [13]. A lower deviation, however, was found by Wittrock et al. [30]. The reason for this finding remains speculative, but might be caused by the repeated measurements of 81 cows resulting in 709 blood samples analysed in the study by Wittrock et al. [30].

The highest deviation with a mean difference of approximately +21 mg/dL for measurements in capillary and +25 mg/dL in coccygeal blood compared with the reference was found for the WGC. It is very likely that the chip’s calibration curve for cats led to this higher deviation. Hence, the development of a cow-specific chip is recommended for this device before further studies in cattle should be conducted.

The determined average inter- and intra-assay coefficients of variation for the FSP (4.2 and 5.2 %) were below 15 % as requested by the European Medicines Agency (EMEA, 2011) for on-site tests. The intra-assay CV of the GLX (14.4 %) and the inter-assay CV of the WGC (12.6 %) were within the upper limit of the requested range, whereas the inter-assay CV of the GLX (19.9 %) and the intra-assay CV of the WGC (15.7 %) exceeded the requested threshold in the bovine species.

The determined correlation coefficients for the glucose concentrations analyzed in capillary blood and the reference test were high, but not excellent for the FSP (73.3 %) and the GLX (80.5 %), whereas the correlation for the WGC was poor (41.2 %). The corresponding results for coccygeal blood were approximately 13 percentage points higher for the FSP, 9 percentage points higher for the WGC, but 3 percentage points lower for the GLX. A possible explanation for the poorer performance of these two devices for measurements in capillary blood might be that sometimes it was necessary to squeeze the skin of the vulva to obtain an adequate amount of blood. Squeezing the skin might cause the excretion of tissue fluid, which mixes up with the blood drop and thereby influences the glucose concentration. This, however, does not explain the smaller difference in the calculated correlation coefficients between capillary and coccygeal blood, respectively, compared with the reference as observed for the other devices. Another possible impact on the results might be the amount of blood applied on the test strips. According to the manufacturer of the WGC, the test strip must remain sufficiently immerged into the blood after the acoustic signal of the device occurs. It should be noticed that this signal indicates the start of the measurement with the WGC, whereas for other devices this signal indicates the end of the measurement procedure.

Bland-Altman plots revealed a negative bias for the FSP and the GLX and a positive bias for the WGC. Glucose concentrations analyzed in plasma and serum are reported to be greater than the corresponding concentrations in whole blood [14, 31]. Kuwa et al. [31] found 13.2 % greater glucose concentrations in plasma compared with those analyzed from venous whole blood. Additionally, significant greater glucose concentrations were found in capillary blood compared with the corresponding concentrations determined in venous blood [31]. This might be a major reason for the observed biases of the FSP and the GLX, but does not explain the differences for the WGC.

With thresholds optimized by ROC analysis, the GLX showed the best performance to detect hyperglycemia using capillary blood as test substrate. The results for the GLX were even better in the capillary blood than in the coccygeal blood. Remarkable lower sensitivities were found for WGC compared with the other devices. The Youden-Index presented in Table 3 confirms that the GLX is the most capable device to analyze glucose concentrations in capillary blood, as this value is the closest to 1, i.e. a ‘perfect test’ [25]. Glucose concentrations determined by the FSP, however, should be interpreted with care, whereas the WGC with the settings for cats is unsuitable.

This study is an investigation of measuring glucose in dairy cows using electronic hand-held devices in capillary and coccygeal blood, respectively. The analyzed glucose concentrations were influenced by the test system and to some extent to the site of sampling. A potential weakness of this study is the composition of the sample set. Samples were collected from animals kept on one commercial farm. Additional field studies are required also to confirm the hypothesized beneficial effects of monitoring the glucose concentration as predictor of the animal’s metabolic status [13], immune competence [3, 5] or reproductive performance [6]. According to the manufacturers manuals the devices deliver reliable measurements for glucose concentrations between 20 and 500 mg/dL (600 mg/dL for the GLX). In our study, the maximum glucose concentration in plasma analyzed at the laboratory was 100 mg/dL. Hence, further studies are necessary to test if the devices lead to reliable results with greater glucose concentrations. Furthermore the validity of clinical decision limits to address hyper- or hypoglycemic statuses, especially for cows during the transition period, has to be assessed in further clinical studies.

## Conclusions

The objective of this study was to test whether capillary blood can be used to determine the glucose concentration in dairy cows using three different electronic hand-held devices. Good test characteristics for determining hyperglycemia by an on-site test were found for the GLX and moderate for the FSP. Hence, these devices are eligible for use in veterinary practice. With the settings for cats, the WGC was not suitable for determination of glucose concentrations neither in capillary nor in coccygeal blood. For this device, the development of a cattle-specific chip is recommended. Capillary blood obtained by minimally invasive puncture of the skin of the exterior vulva is considered as suitable for analyzing glucose concentrations.

## Abbreviations

AUC: area under the receiver operating characteristics curve
BHBA: ß-hydroxybutyric acid
CCPU: Central Clinical Pathology Unit
CV: coefficient of variation
FSP: FreeStyle Precision
GLX: GlucoMen LX Plus
IR: insulin resistance
*J*: Youden-Index
MC: MiniCollect safety lancet
MT: Microtainer contact-activated lancet
PMN: polymorphnuclear neutrophils
ROC: receiver operating characteristics
SE: sensitivity
SL: SafetyLancets special
SP: specificity
WGC: WellionVet GLUCO CALEA
